# The Therapeutics of the Ichthyol Compounds

**Published:** 1904-06-11

**Authors:** 


### THE THERAPEUTICS OF THE ICHTHYOL COMPOUNDS.

By combination with various active therapeutic substances such preparations as iron-ichthyol, sodium-ichthyol, etc., can be formed, and many of these have considerable value as medicinal agents. Iron-ichthyol contains 3·5 per cent. of iron. It is best administered in cachets or tablets (5 to 10 grs. thrice daily), and though not effective in chlorosis, is of great value in secondary anæmias, as after rheumatism and in renal disease. Unna advises it in chronic skin affections associated with anæmia. Sodium-ichthyol may be presented in pill form, and is used to check fermentation in gastric catarrh. For pulmonary diseases it is less valuable than the ammonia compound. Ichthyform is a combination of ichthyol with formic aldehyde, and in the presence of alkalies splits up into its two components; it thus has astringent and antiseptic properties. It is a tasteless, odourless, insoluble powder, and is non-toxic. In the summer diarrhœa of infants, and in tuberculous lesions of the intestine, it has given good results. Infants may take 2-grain doses in jelly every six hours; in older children the dose may be increased to 10 or 15 grains. Polacco, of Milan, has used it in enteric fever, giving half-gramme doses frequently. Ichthyform is a valuable application to varicose and foul ulcers, and where there are redundant granulations it is well to use it with an equal bulk of zinc oxide. For dysidrosis equal parts of bismuth, starch, and ichthyform form a useful combination. In subacute and chronic eczemas ichthyform ointment (1 to 5 per cent. with vaseline) is highly successful. Ichthyform gauze is an effective substitute for iodoform gauze, and is a more economical dressing.

Another compound of ichthyol is ichthargan, a silver salt of ichthyol. This also is an amorphous, odourless powder, freely soluble in water. It has greater germicidal action than silver nitrate on gonococci and other micro-organisms. As an injection in gonorrhœa it may be used in the strength of 1 in 5,000 to 1 in 500, commencing with the weaker solution. In leucorrhœa a 10 per cent. solution in glycerine applied on tampons is useful, but the best results are obtained by pessaries containing from ½ to 1 grain. One of these is placed in the posterior vaginal fornix every alternate night and followed by boric acid douches on the following day. In dermatological practice an ointment of 1 to 2 per cent. (with lanoline and vaseline, equal parts) is most generally applicable, but to abort boils and in ringworm of the scalp a 5 to 10 per cent. ointment is needed. In atrophic rhinitis ichthargan acts as a deodorant and also as an anti-inflammatory agent; a 4 per cent. solution should be used. Simple or specific laryngitis is well treated by a pigment of 5 per cent. strength. Ichthargan has also been used in blepharitis and ophthalmia, but there is no reason to believe that here it will supplant some of the older methods.

[1] Jas. Burnet, M.B., Lancet, March 12, 1904.

